# Telehealth vs In-Clinic Medication Abortion Services

**DOI:** 10.1001/jamanetworkopen.2023.31900

**Published:** 2023-09-01

**Authors:** Anna E. Fiastro, Zihan Zheng, Molly R. Ruben, Jessica Gipson, Emily M. Godfrey

**Affiliations:** 1 Department of Family Medicine, University of Washington, Seattle; 2 Department of Community Health Sciences, Fielding School of Public Health, University of California, Los Angeles

## Abstract

This cross-sectional study compares differences in patient characteristics between those using telehealth vs in-clinic services for medication abortion care.

## Introduction

Abortion is a common health care service, yet federal and state regulations continue to restrict care options, with disproportionate impacts on underserved communities.^1,2,3^ Due to limited in-clinic access throughout the country, telehealth medication abortion services (tele-MAB), including online consultations and medications delivered directly to patients, can make abortion care more convenient and accessible.^4^

Other areas of health care have documented disparities in telehealth access, although less is known about tele-MAB services.^5^ We sought to understand differences in patient characteristics between those using telehealth vs in-clinic for medication abortion care and to explore the role of tele-MAB in serving diverse patient populations.

## Methods

This cross-sectional study follows the STROBE reporting guideline^6^ and was approved by the University of Washington and University of California, Los Angeles institutional review boards with exemption from informed consent because data were deidentified in accordance with 45 CFR § 46. We analyzed electronic medical record data from tele-MAB and tele-MAB–eligible patients who received in-clinic medication abortion services at Cedar River Clinics, a reproductive health care clinic in Washington State, between April 23, 2020, and January 31, 2022 (eFigure in Supplement 1). A sensitivity analysis was completed excluding in-clinic patients with missing information on tele-MAB eligibility (208 patients [17%]). Using bivariable and multivariable logistic regressions, we examined associations of patient characteristics (eTable in Supplement 1) with odds of receiving tele-MAB vs in-clinic services. We used 2-sided tests with an α criterion of .05, using Stata IC statistical software version 16.1 (StataCorp). Data analysis was conducted from September to December 2022.

## Results

Of the 1241 patients (mean [range] age, 30 [13-52] years) included in the study, 858 individuals (69%) received in-clinic appointments for medication abortion services and 383 (31%) received tele-MAB services (Table 1). In the unadjusted analysis, number of living children and payment type were not different between tele-MAB and in-clinic patients. In the adjusted model, individuals who lived further from a clinic and had at least 1 prior abortion were more likely to receive tele-MAB (Table 2). Compared with White individuals, patients who self-reported as multiracial or other race were also more likely to receive tele-MAB, whereas Black individuals were more likely to be seen in-clinic. Younger individuals, non-English speakers, and those with at least 1 health condition were also less likely to receive tele-MAB vs in-clinic services. The association of gestational duration (GD) with appointment type was not significant in the multivariable model but was significant in the sensitivity analysis, with those at 57 to 70 days GD more likely to choose tele-MAB than those at 43 to 56 days (adjusted odds ratio, 1.75; 95% CI, 1.06-2.89). The sensitivity analysis found all other associations to be robust.

**Table 1.** Description of Study Sample

Patient demographic characteristic	Patients receiving medication abortion services, April 23, 2020 to January 31, 2022, No. (%)			*P* value^a^
	Total (N = 1241)	In-clinic (n = 858)	Telehealth (n = 383)	
Age, y				
Mean, range	30 (13-52)	30 (13-52)	31 (15-47)	.002
<20	92 (7.4)	63 (7.3)	29 (7.6)	
21-25	244 (19.7)	193 (22.5)	51 (13.3)	
26-30	339 (27.3)	236 (27.5)	103 (26.9)	
31-35	312 (25.1)	201 (23.4)	111 (29.0)	
≥36	254 (20.5)	165 (19.2)	89 (23.2)	
Self-declared gender				
Women	1209 (97.4)	841 (98.0)	368 (96.1)	.08
Men	1 (0.1)	1 (0.1)	0	
Missing data	31 (2.5)	16 (1.9)	15 (3.9)	
Self-declared race and ethnicity				
Asian, Native Hawaiian, or Other Pacific Islander	220 (17.7)	246 (18.8)	53 (13.8)	<.001
Black or African American	232 (18.7)	273 (20.9)	46 (12.0)	
Hispanic or Latino	179 (14.2)	206 (15.8)	53 (13.8)	
White	361(29.1)	387 (29.6)	115 (30.0)	
Multiracial or other race	150 (12.1)	77 (5.9)	98 (25.6)	
Declined to specify or no response	102 (8.2)	117 (9.0)	18 (4.7)	
Primary language				
English	1163 (93.7)	782 (91.1)	381 (99.5)	<.001
Other	78 (6.3)	76 (8.9)	2 (0.5)	
Living children				
Mean, range	1 (0-7)	1 (0-7)	1 (0-7)	.22
0	472 (38.0)	339 (39.5)	133 (34.7)	
≥1	743 (59.9)	509 (59.3)	234 (61.1)	
Missing data	26 (2.1)	10 (1.2)	16 (4.2)	
Type of payment for visit				
Cash self-pay	279 (22.5)	179 (20.9)	100 (26.1)	.12
Public insurance	595 (48.0)	419 (48.8)	176 (46.0)	
Private insurance	367 (29.6)	260 (30.3)	107 (27.9)	
Social Vulnerability Index rank				
Low	889 (71.6)	635 (74.0)	254 (66.3)	<.001
Medium	316 (25.5)	211 (24.6)	105 (27.4)	
High	33 (2.7)	9 (1.1)	24 (6.3)	
Missing	3 (0.2)	3 (0.4)	0	
Distance to Cedar River Clinic locations, mi				
<5	267 (21.5)	203 (23.6)	64 (16.7)	<.001
5-10	410 (33.0)	300 (35.0)	110 (28.7)	
10-25	428 (34.5)	294 (34.3)	134 (35.0)	
25-50	73 (5.9)	44 (5.1)	29 (7.6)	
>50	55 (4.4)	12 (1.4)	43 (11.2)	
Missing	8 (0.6)	5 (0.6)	3 (0.8)	
Past and current health conditions				
Mean, range	1 (0-14)	1 (0-14)	0 (0-9)	<.001
0	790 (63.7)	489 (57.0)	301 (78.6)	
1	194 (15.6)	164 (19.1)	30 (7.8)	
≥2	227 (18.3)	196 (22.8)	31 (8.1)	
Missing data	30 (2.4)	9 (1.1)	21 (5.5)	
Prior abortions				
Mean, range	0.87 (0-11)	0.87 (0-11)	0.85 (0-9)	.06
0	691 (55.7)	499 (58.2)	192 (50.1)	
≥1	521 (42.0)	350 (40.8)	171 (44.7)	
Missing data	29 (2.3)	9 (1.1)	20 (5.2)	
Gestational duration, d				
Mean, range	45 (13-70)	45 (22-70)	46 (13-70)	.08
≤42	521 (42.0)	370 (43.1)	151 (41.0)	
43-56	535 (43.1)	378 (44.1)	157 (39.4)	
57-70	152 (12.3)	94 (11.0)	58 (15.1)	
Missing data	33 (2.7)	16 (1.9)	17 (4.4)	
Eligibility for telehealth medication abortion and patient was asked to come into clinic				
Eligible	375 (30.2)	NA	375 (97.9)	NA
Ineligible	8 (<0.1)	NA	8 (2.1)	

Abbreviation: NA, not applicable.

^a^ *P* values were calculated using χ^2^ tests.

**Table 2.** Unadjusted and Adjusted ORs for Receiving Tele-MAB Compared With In-Clinic Medication Abortion Services

Patient characteristic	Likelihood of receiving tele-MAB vs in-clinic medication abortion services, OR (95% CI)			
	Unadjusted model (N =1241)	*P* value^a^	Adjusted multivariable model (n = 1156)^b^	*P* value^a^
Age, y				
<20	0.83 (0.51-1.37)	.003	0.49 (0.26-0.92)^c^	<.001
21-25	0.48 (0.33-0.70)^d^		0.35 (0.22-0.57)^d^	
26-30	0.79 (0.57-1.10)		0.60 (0.40-0.90)^c^	
31-35	1 [Reference]		1 [Reference]	
≥36	0.98 (0.69-1.38)		0.9 (0.64-1.4)	
Self-declared race and ethnicity				
Asian, Native Hawaiian, or Other Pacific Islander	0.68 (0.46-1.36)^c^	<.001	0.87 (0.56-1.37)	<.001
Black or African American	0.53 (0.36-0.78)^e^		0.61 (0.39-0.95)^c^	
Hispanic or Latino	0.92 (0.62-1.36)		0.88 (0.5-1.43)	
White	1 [Reference]		1 [Reference]	
Multiracial or other race	4.03 (2.70-6.03)^c^		4.52 (2.78-7.34)^c^	
Declined to specify or no response	0.46 (0.26-0.80)^e^		0.476(0.25-0.88)^c^	
Preferred language				
English	1 [Reference]	<.001	1 [Reference]	<.001
Other	0.05 (0.01-0.22)^d^		0.06 (0.02-0.26)^e^	
No. of living children				
0	1 [Reference]	.22	NA	NA
≥1	1.17 (0.91-1.51)		NA	
Type of payment for visit				
Public insurance	1 [Reference]	.12	NA	NA
Private insurance	0.98 (0.74-1.30)		NA	
Cash self-pay	1.33 (0.98-1.80)		NA	
Social Vulnerability Index rank				
Low	1 [Reference]	<.001	1 [Reference]	.70
Medium	1.24 (0.94-1.64)		1.14 (0.81-1.61)	
High	6.67 (3.06-14.5)^d^		1.31 (0.37-4.69)	
Distance to Cedar River Clinic location, mi	1.02 (1.02-1.03)^d^	<.001	1.02 (1.01-1.03)^d^	<.001
Past and current health conditions, No.				
0	1 [Reference]	<.001	1 [Reference]	<.001
1	0.30 (0.20-0.45)^c^		0.24 (0.15-0.38)^c^	
≥2	0.26 (0.17-0.39)^d^		0.19 (0.12-0.31)^d^	
Prior abortions, No.				
0	1 [Reference]	.058	1 [Reference]	.002
≥1	1.27 (0.99-1.63)		1.63 (1.20-2.22)^e^	
Gestational duration, d				
≤42	0.98 (0.75-1.28)	.08	0.83 (0.60-1.13)	.22
43-56	1 [Reference]		1 [Reference]	
57-70	1.49 (1.02-2.16)^c^		1.21 (0.77-1.90)	

Abbreviations: NA, not available; OR, odds ratio; tele-MAB, telehealth medication abortion services.

^a^ Reported *P* values from Wald test.

^b^ Variables statistically significant in the χ^2^ bivariate analyses (*P* < .10) were included in the multivariable model. For the multivariable model, 85 observations were dropped due to missingness (5 for distance and 80 missing on multiple covariates).

^c^ *P* < .05.

^d^ *P* < .001.

^e^ *P* < .01.

## Discussion

Our findings suggest that tele-MAB facilitates abortion care access for those further from brick-and-mortar abortion facilities and, thus, may mitigate the impacts of travel logistics and costs. Additionally, tele-MAB may better meet the needs of those with prior abortion experience, perhaps due to greater familiarity with the abortion process. Further research is needed to better understand patients who identified as multiracial or other race and how identity, including prior experiences of racism in health care settings, may impact patient preference for tele-MAB services. Additional research is also needed to determine whether those at later GD are more likely to choose tele-MAB, perhaps anticipating initiation of their termination.

Our findings also suggest some groups with limited access to abortion care (ie, younger individuals, those with limited English proficiency, and those with health conditions) are less likely to use tele-MAB services. Efforts to educate patients about telehealth procedures and improve availability of language support are needed to make tele-MAB feasible for diverse populations. Limitations of this study are that findings may not be generalizable to asynchronous or less-integrated telemedicine models, or other care settings.

Patients face many barriers to accessing abortion care, and tele-MAB services facilitate more convenient and timely care. Tele-MAB services reach some communities traditionally underserved by clinic-based services, addressing some disparities in receipt of care. As access to abortion care continues to be restricted in the US, innovative models of care delivery are needed to accommodate the sustained demand for services and to meet the needs of diverse patient populations.

## Notes

Supplement 1. **eFigure.** Description of Eligible Study Sample From All Who Received Medication Abortion Services During the Study Period (April 23, 2020 – January 31, 2022) **eTable.** Definitions of Patient Characteristics
eReferences

Supplement 2.
Data Sharing Statement

## References

[1] Jones RK, Jerman J. Population group abortion rates and lifetime incidence of abortion: United States, 2008-2014. Am J Public Health. 2017;107(12):1904-1909. doi:10.2105/AJPH.2017.304042 29048970 PMC5678377

[2] Gerdts C, Fuentes L, Grossman D, . Impact of clinic closures on women obtaining abortion services after implementation of a restrictive law in Texas. Am J Public Health. 2016;106(5):857-864. doi:10.2105/AJPH.2016.303134 26985603 PMC4985084

[3] Upadhyay UD, Johns NE, Cartwright AF, Franklin TE. Sociodemographic characteristics of women able to obtain medication abortion before and after Ohio’s law requiring use of the Food and Drug Administration protocol. Health Equity. 2018;2(1):122-130. doi:10.1089/heq.2018.0002 30283858 PMC6071907

[4] Fiastro AE, Sanan S, Jacob-Files E, . Remote delivery in reproductive health care: operation of direct-to-patient telehealth medication abortion services in diverse settings. Ann Fam Med. 2022;20(4):336-342. doi:10.1370/afm.2821 35831175 PMC9328706

[5] Reed ME, Huang J, Graetz I, . Patient characteristics associated with choosing a telemedicine visit vs office visit with the same primary care clinicians. JAMA Netw Open. 2020;3(6):e205873. doi:10.1001/jamanetworkopen.2020.5873 32585018 PMC7301227

[6] von Elm E, Altman DG, Egger M, Pocock SJ, Gøtzsche PC, Vandenbroucke JP; STROBE Initiative. The strengthening the reporting of observational studies in epidemiology (STROBE) statement: guidelines for reporting observational studies. J Clin Epidemiol. 2008;61(4):344-349. doi:10.1016/j.jclinepi.2007.11.008 18313558

