# Error in Results

**DOI:** 10.1001/jamanetworkopen.2023.38104

**Published:** 2023-09-22

In the Research Letter titled “Telehealth vs In-Clinic Medication Abortion Services,”^1^ published September 1, 2023, there were numerical errors in Table 1 and Table 2 reported in the Results. In Table 1, the total number of Hispanic or Latino participants should be 176, and numbers and percentages were incorrect for all race and ethnicity categories for in-clinic abortions. In Table 2, the odds ratio in the adjusted multivariable model for participants aged 36 years or older should be 0.98, and the 95% CI in the unadjusted model for Asian, Native Hawaiian, or Other Pacific Islander participants should be 0.46 to 0.99. This article has been corrected.^1^
