# Supplementary material for: Telehealth vs In-Clinic Medication Abortion Services
Source: JAMA Netw Open. 2023 Sep 1;6(9):e2331900. doi: 10.1001/jamanetworkopen.2023.31900 (PMC10474522; doi:10.1001/jamanetworkopen.2023.31900)
Supplement: Supplement 1. — eFigure. Description of Eligible Study Sample From All Who Received Medication Abortion Services During the Study Period (April 23, 2020 – January 31, 2022) eTable. Definitions of Patient Characteristics eReferences [file jamanetwopen-e2331900-s001.pdf]

Supplemental Online Content

Fiastro AE, Zheng Z, Ruben MR, Gipson J, Godfrey EM. *JAMA Netw Open*. 2023;6(9):e2331900. doi:10.1001/jamanetworkopen.2023.31900

eFigure. Description of Eligible Study Sample From All Who Received Medication Abortion Services During the Study Period (April 23, 2020 – January 31, 2022).

eTable. Definitions of Patient Characteristics

eReferences

This supplemental material has been provided by the authors to give readers additional information about their work.

© 2023 Fiastro AE et al. *JAMA Network Open.*

eFigure. Description of Eligible Study Sample From All Who Received Medication Abortion Services During the Study Period (April 23, 2020 – January 31, 2022).

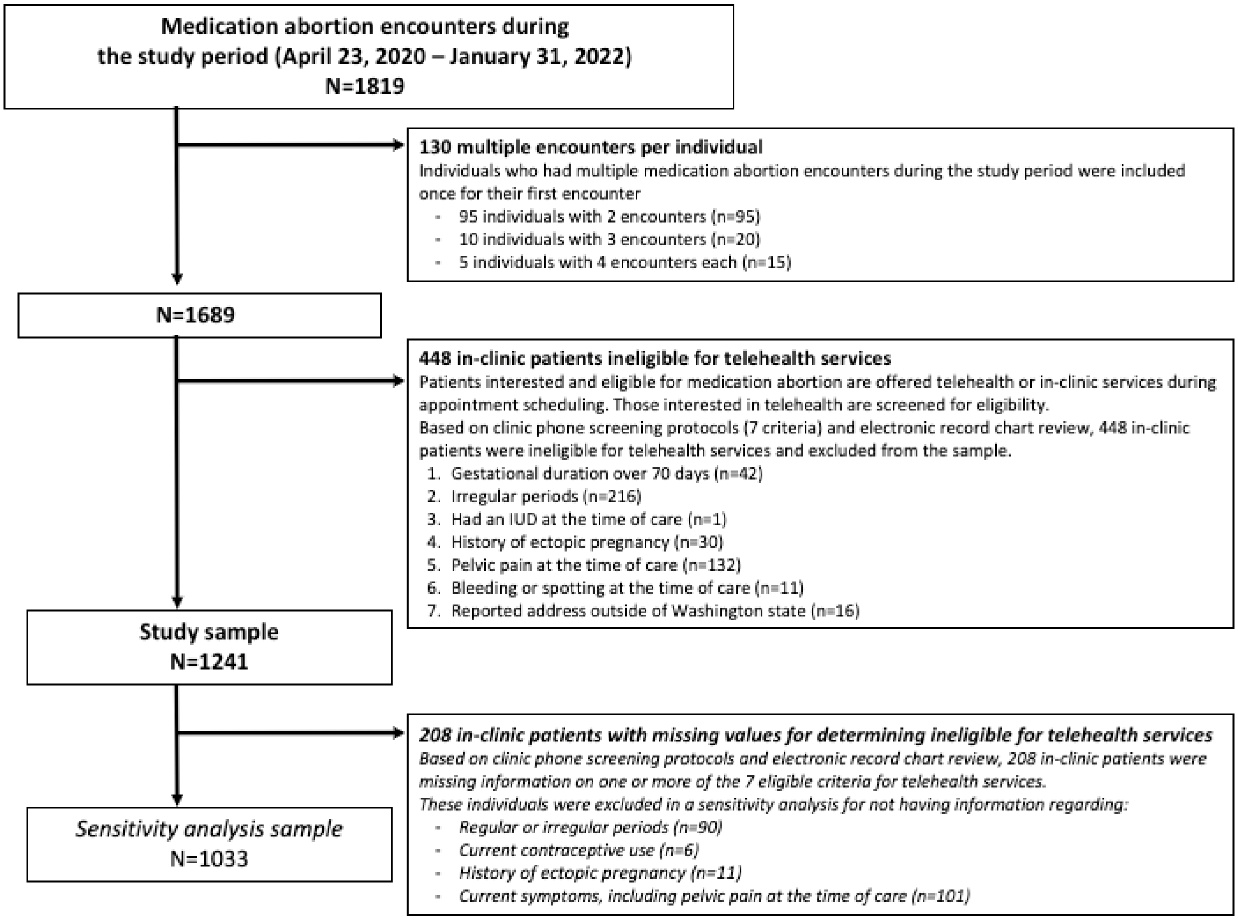

© 2023 Fiastro AE et al. *JAMA Network Open.*

eTable. Definitions of Patient Characteristics

**Measure**	**Description**
Age	Patient age was calculated in years on the date of the appointment using the patient's date of birth.
Gender and Race/ethnicity	Race and ethnicity were examined due to documented disparities in telehealth access and utilization. Patients self-identified their gender, race and ethnicity when completing the patient history intake form. All individuals who identified as Hispanic or Latino were included as such, those who identified as Non-Hispanic or declined to specify were included based on their self-selected race. 'Asian' was combined with "Native Hawaiian or Other Pacific Islander"; 'Alaska Native', 'American Indian', 'more than one race', 'multi-racial', and 'other race' were combined creating 'Multi-racial / Other race'; 'Declined to specify' and those who did not respond were combined; those who chose 'Black or African American' and 'White' were included as such.
Language	When scheduling an appointment, patients were given the option to complete their consultation in a language other than English with an interpreter regardless of whether their appointment was via telehealth or in-person. Those who chose to speak English were included as such. Patients who had their appointment with a translator in another languages (23 languages) were included as non-English speakers.
Past and current health conditions	Patients indicated current experience or history of 47 common health issues or 'No known health issues' in the patient history intake form. None of these health issues were contraindications for telehealth or medication abortion services. Individuals were categorized as having no health issues, one, or two or more if they selected any health issue(s).
Payer type	Patients had the same payment options regardless of whether they chose telehealth or in-person services. Payer type is the method used by the patient to pay for their services, categorized by the clinic's financial staff as: cash self-payment (no insurance used), public insurance, or private insurance plan (mutually exclusive).
Gestational duration	Gestational duration (GD) was documented by clinic staff using ultrasound or calculated using reported last menstrual period and consultation date. Patient charts with GD above 70 days were reviewed for additional information. Those still missing GD or with a GD over 70 days were included as missing GD based on clinic protocols (n=33); most had documented pregnancies of unknown location.
Social vulnerability	Using the Housing and Urban Development United States Postal Service (HUD USPS) zip code and the United States Department of Agriculture

© 2023 Fiastro AE et al. *JAMA Network Open.*

	county name crosswalk files, we matched patient-reported zip code with specific counties using associated Federal Information Processing Standards codes.[1] For zip codes that matched multiple Federal Information Processing Standard count codes, we used the primary county for a given zip code. We then assigned county-level Centers for Disease Control and Prevention Social Vulnerability Index (CDC SVI) scores.[2] The CDC SVI is a 15-factor metric that includes poverty, lack of access to transportation, adequate housing, and minority status and language. The CDC SVI was chosen due to its comprehensive inclusion of social determinants of health at the neighborhood level and its use to examine the relationship between social vulnerability and a wide range of health behaviors and outcomes.[3-6] We categorized patient social vulnerability into terciles of low, medium, and high.
Distance to Cedar River Clinic locations	Using the U.S. Department of Housing and Urban Development's zip code population weighted centroid point, we assigned a latitude/longitude point to each individual based on the zip code the patient reported when scheduling and took the shortest of the two distances in miles between the patient's location and Cedar River Clinics Renton and Tacoma clinical locations using Vincenty's formula.[7,8] One telehealth patient reported an address in Florida which was included as missing since CRC does not serve telehealth patients unless they are in Washington state.

© 2023 Fiastro AE et al. *JAMA Network Open.*

eReferences

1. HUD USPS zip code crosswalk files. HUD User Office of Policy Development and Research. Accessed March 1, 2021. https://www.huduser.gov/portal/datasets/usps_crosswalk.html#data
2. CDC/ATSDR social vulnerability index. Agency for Toxic Substances and Disease Registry. Accessed February 15, 2021. https://www.atsdr.cdc.gov/placeandhealth/svi/index.html
3. Diaz A, Hyer JM, Azap R, Tsilimigras D, Pawlik TM. Association of social vulnerability with the use of high-volume and Magnet recognition hospitals for hepatopancreatic cancer surgery. *Surgery*. 2021;170(2):571-578. doi:10.1016/j.surg.2021.02.038
4. Morgan ME, Horst MA, Vernon TM, et al. An analysis of pediatric social vulnerability in the Pennsylvania trauma system. *J Pediatr Surg*. 2020;55(12):2746-2751. doi:10.1016/j.jpedsurg.2020.05.024
5. Biggs EN, Maloney PM, Rung AL, Peters ES, Robinson WT. The relationship between social vulnerability and COVID-19 incidence among Louisiana census tracts. *Front Public Health*. 2021;8. doi:10.3389/fpubh.2020.617976
6. Yap A, Laverde R, Thompson A, et al. Social vulnerability index (SVI) and poor postoperative outcomes in children undergoing surgery in California. *Am J Surg*. 2022. doi:10.1016/j.amjsurg.2022.09.030
7. Zip code population weighted controls. HUD User Office of Policy Development and Research. Published December 31, 2020. Accessed December 6, 2022. https://hudgis-hud.opendata.arcgis.com/datasets/HUD::zip-code-population-weighted-centroids/about
8. Nichols A. *VINCENTY: stata module to calculate distances on the earth's surface*. Statistical Software Components S456815, Boston College Department of Economics; revised February 16, 2007. Accessed December 6, 2022. https://ideas.repec.org/c/boc/bocode/s456815.html

© 2023 Fiastro AE et al. *JAMA Network Open.*
