# Supplementary material for: Telehealth vs In-Clinic Medication Abortion Services
Source: JAMA Netw Open. 2023 Sep 1;6(9):e2331900. doi: 10.1001/jamanetworkopen.2023.31900 (PMC10474522; doi:10.1001/jamanetworkopen.2023.31900)
Supplement: Supplement 2. — Data Sharing Statement [file jamanetwopen-e2331900-s002.pdf]

Data Sharing Statement

Fiastro. Telehealth vs In-Clinic Medication Abortion Services. *JAMA Netw Open*. Published September 01, 2023. doi:10.1001/jamanetworkopen.2023.31900

Data
Data available: No

Additional Information
Explanation for why data not available: Patient privacy given stigmatized nature of abortion care
